# Associations between national viral hepatitis policies/programmes and country-level socioeconomic factors: a sub-analysis of data from the 2013 WHO viral hepatitis policy report

**DOI:** 10.1186/s12889-017-4549-4

**Published:** 2017-07-26

**Authors:** Jeffrey V Lazarus, Ida Sperle, Kelly Safreed-Harmon, Charles Gore, Beatriz Cebolla, Alexander Spina

**Affiliations:** 1 CHIP, Department of Infectious Diseases, Rigshospitalet, University of Copenhagen, Oester Allé 56, 5th Floor, DK-2100 Copenhagen, Oe Denmark; 2 Barcelona Institute for Global Health (ISGlobal), Hospital Clínic, University of Barcelona, Barcelona, Spain; 3 World Hepatitis Alliance, London, UK; 4 Independent consultant, Cologne, Germany; 5 Centre for Infectious Disease Epidemiology, Austrian Agency for Health and Food Safety (AGES), Vienna, Austria; 6 European Programme for Intervention Epidemiology Training (EPIET), European Centre for Disease Prevention and Control (ECDC), Stockholm, Sweden

**Keywords:** Health policy, Surveillance, Viral hepatitis

## Abstract

**Background:**

As more countries worldwide develop national viral hepatitis strategies, it is important to ask whether context-specific factors affect their decision-making. This study aimed to determine whether country-level socioeconomic factors are associated with viral hepatitis programmes and policy responses across WHO Member States (MS).

**Methods:**

WHO MS focal points completed a questionnaire on national viral hepatitis policies. This secondary analysis of data reported in the *2013 Global Policy Report on the Prevention and Control of Viral Hepatitis in WHO Member States* used logistic regression to examine associations between four survey questions and four socioeconomic factors: country income level, Human Development Index (HDI), health expenditure and physician density.

**Results:**

This analysis included 119 MS. MS were more likely to have routine viral hepatitis surveillance and to have a national strategy and/or policy/guidelines for preventing infection in healthcare settings if they were in the higher binary categories for income level, HDI, health expenditure and physician density. In multivariable analyses, the only significant finding was a positive association between having routine surveillance and being in the higher binary HDI category (adjusted odds ratio 26; 95% confidence interval 2.0–340).

**Conclusion:**

Countries with differing socioeconomic status indicators did not appear to differ greatly regarding the existence of key national policies and programmes. A more nuanced understanding of the multifaceted interactions of socioeconomic factors, health policy, service delivery and health outcomes is needed to support country-level efforts to eliminate viral hepatitis.

## Background

Hepatitis B virus (HBV) and hepatitis C virus (HCV) combined are the seventh-leading cause of death globally, with most deaths resulting from HBV- and HCV-associated cirrhosis and liver cancer [1]. There are thought to be approximately 250 million people worldwide living with chronic HBV infection [2], while the chronically HCV-infected population worldwide is thought to number approximately 130–150 million [3]. Despite regional variations, viral hepatitis is widely regarded as a global public health problem. In 2013, low-income and lower-middle-income countries accounted for 42% of viral hepatitis deaths, and upper-middle-income and high-income countries, 58% [1].

There are reliable interventions for preventing and managing both infections, including a safe and effective hepatitis B vaccine [4] as well as antiviral treatment regimens that can cure more than 90% of cases of hepatitis C [3]. Although HBV cannot be cured, the damaging consequences of the virus often can be greatly reduced through medical interventions [4]. In light of the current state of knowledge, it is theoretically possible to eliminate both diseases from all populations worldwide. However, the current global disease burden suggests that many countries are far from achieving sufficient uptake of strategies proven to work well.

The inclusion in the Sustainable Development Goals of a target to “combat hepatitis” [5] reflects a growing consensus that this group of diseases, particularly HBV and HCV, must be addressed decisively at the global and national policy levels. The World Health Assembly’s 2010 and 2014 viral hepatitis resolutions [6, 7] have given direction to the policy response, in part by informing how the World Health Organization (WHO) works with Member States (MS) on viral hepatitis issues. More recently, MS endorsed WHO’s goal of eliminating viral hepatitis as a public health threat with their approval of the *Global Health Sector Strategy on Viral Hepatitis, 2016–2021* [3].

As of this writing, the only published assessment of national policy responses to viral hepatitis across multiple countries and regions is the *2013 Global Policy Report on the Prevention and Control of Viral Hepatitis in WHO Member States* [8]. Survey findings presented in the report indicate that at the time the data were collected, WHO MS diverged from each other in many ways regarding how they addressed viral hepatitis. In the current policy environment, when increasingly more countries worldwide are developing their first national viral hepatitis strategies or considering how to strengthen existing strategies, it is important to try to identify context-specific factors that may affect their decision-making.

Implementing the policy measures advocated by the World Health Organization in the *Global Health Sector Strategy* and in its guidance on the development of national viral hepatitis plans [9] requires multiple types of resources such as financial resources, technical expertise and an extensive health system infrastructure. Socioeconomic indicators relating to country income level, Human Development Index (HDI) score, health expenditure and physician density have been investigated as possible predictors of maternal mortality and the incidence and prevalence of cancer, infectious diseases and injuries, among other health outcomes [10–14]; this raises the question of whether such proxy measures of how well-resourced a country is can also be used to predict a strong or weak policy response to viral hepatitis. The following study sought to identify associations between country-level socioeconomic indicators and viral hepatitis programme and policy responses across WHO MS.

## Methods

### Data sources

The methodology for the survey that provided the basis of the *2013 Global Policy Report* has been described elsewhere [8]. In short, a survey with 43 policy-related questions was sent to the government focal point for viral hepatitis in each of the 194 MS of the World Health Organization. Data collection took place from July 2012 to February 2013.

Four survey questions were selected from the *2013 Global Policy Report* as measures of Member States’ commitment to combatting viral hepatitis. These served as dependent variables for analyses performed in this study. One question asked if countries have a written national strategy or plan that focuses exclusively or primarily on the prevention and control of viral hepatitis. The second question asked if there is routine surveillance for viral hepatitis. The third question asked if governments had funded World Hepatitis Day or any public viral hepatitis awareness campaign since January 2011. The fourth question asked if there is a national strategy and/or policy/guidelines for preventing hepatitis B and hepatitis C infection in healthcare settings.

A follow-up question to the fourth question asked if health workers were vaccinated against hepatitis B prior to starting work that might put them at risk of exposure to blood.

The four socioeconomic indicators chosen to serve as independent variables in the current study came from multiple sources. Income level data were drawn from the World Bank country classifications for 2013, which were defined as follows: low income, ≤$1035; lower middle income, $1036–$4085; upper middle income, $4086–$12,615; and high income, ≥$12,616 [15]. HDI data for 2013 were sourced from the United Nations Development Programme (UNDP) database, which provides a proxy index of development using the geometric mean of three measures: life expectancy at birth, mean years over expected years of schooling, and gross national income (GNI) per capita in US dollars based on purchasing power parity [16]. Health expenditure data for 2013 were obtained from the WHO global health expenditure database, which reflects per capita total expenditure on health in US dollars at the average exchange rate [17]. Physician density, as indicated by the number of medical doctors per 1000 people, was obtained from the WHO global health workforce statistics database [18]. Data were for 2013 or the most recent year prior to that for which information was available.

### Data analysis

WHO MS that submitted surveys for the *2013 Global Policy Report* were included in the current study if they had data available for all four independent variables considered in this study. Each Member State received a binary designation of “low” or “high” for each independent variable as well as a quadripartite designation of “low”, “lower middle”, “upper middle” or “high”. The categories for country income level were defined in accordance with the four World Bank income categories [19], with the lower two World Bank groupings constituting the “low” group and the higher two constituting the “high” group for the binary classification. The categories for the other three independent variables were determined by calculating quartiles for each dataset, i.e., for HDI scores, health expenditure and physician density. In each case, quartiles were determined using values from the full dataset rather than from only the countries included in the study analysis.

Proportions were calculated using the number of responding MS as denominators. Proportions were compared using the Fisher’s exact test. The Mann-Whitney test was used with quadripartite groupings. Univariate logistic regression was used to calculate odds ratios (OR) and 95% confidence intervals (CI) for the four binary independent variables in relation to each dependent variable. All possible combinations of independent variables were used in logistic regressions with each dependent variable in order to test whether any associations were affected by independent variables influencing each other (confounding, effect modification and collinearity). Univariate logistic regression with quadripartite independent variables was used to investigate stepwise relationships for each of the dependent variables. The lowest category was the reference group in each case. Binary independent variables were selected for the multivariable models based on univariate results. Final models for each of the dependent variables were selected based on stepwise forward multivariable logistic regression using the Akaike information criterion and likelihood ratio tests.

All analyses were two-tailed, with a significance level of 0.05. All analyses were carried out using R statistical software version 3.1.2 (Foundation for Statistical Computing, Vienna, Austria).

## Results

### Respondents

The survey that provided the basis for the *2013 Global Policy Report* was completed by 126 of 194 WHO Member States (65%). Data for one or more of the independent variables considered in this study were not available for seven MS (Democratic People’s Republic of Korea, Russian Federation, San Marino, Slovenia, Somalia, South Sudan and Zimbabwe). Thus 119 MS – 61% of all MS – were included in the analysis.

The response rate was higher for MS with HDI scores in the above-median category in comparison to below-median countries (*p* = 0.001). Likewise, MS with above-median physician density had higher response rates than those with below-median physician density (*p* < 0.001) (data not shown).

### Descriptive analysis

Among the 119 MS included in the analysis, 44 (37%) reported having a written national viral hepatitis strategy or plan. Ninety-nine (83%) reported having routine surveillance for viral hepatitis; 53 (45%) reported funding viral hepatitis awareness campaigns; 84 (71%) reported having a strategy and/or policy/guidelines for preventing hepatitis B and hepatitis C infection in healthcare settings; and 77 (79%) reported that health workers were vaccinated against hepatitis B prior to starting work that might put them at risk of exposure to blood.

Significantly higher proportions of Member States reported having routine surveillance, having a healthcare setting prevention strategy and/or policy/guidelines, and having health worker vaccination in the higher binary categories for all four socioeconomic indicators. The proportions of affirmative survey answers for the same three dependent variables also increased with each increase in quartile categories for all four socioeconomic indicators. Mann-Whitney tests showed statistically significant increasing trends in each case (Table 1). For the survey question about routine viral hepatitis surveillance, 100% of respondents in the highest quartiles for all four socioeconomic indicators reported having routine surveillance.

**Table 1:** Frequency and proportion of respondents reporting policies or programme activities by socioeconomic indicator quartiles

	Income level groups *n* (% group)				
	Low *N* = 14	Lower middle *N* = 34	Upper middle *N* = 33	High *N* = 38	*p*-value^ǂ^
National strategy or plan	4 (29)	13 (38)	13 (39)	14 (37)	<0.001
Routine surveillance	6 (43)	25 (74)	30 (91)	38 (100)	<0.001
Awareness campaign	3 (21)	18 (53)	17 (52)	15 (40)	<0.001
Prevention in healthcare settings	5 (36)	17 (50)	29 (88)	33 (87)	<0.001
Health worker vaccination	2 (25)	15 (60)	27 (87)	33 (97)	<0.001
	Human Development Index groups *n* (% group)				
	Low *N* = 21	Lower middle *N* = 28	Upper middle *N* = 33	High *N* = 37	*p*-value^ǂ^
National strategy or plan	4 (19)	14 (50)	12 (36)	14 (38)	<0.001
Routine surveillance	8 (38)	22 (79)	32 (97)	37 (100)	<0.001
Awareness campaign	8 (38)	12 (43)	16 (49)	17 (46)	<0.001
Prevention in healthcare settings	7 (33)	17 (61)	26 (79)	34 (92)	<0.001
Health worker vaccination	2 (20)	16 (64)	25 (89)	34 (97)	<0.001
	Health expenditure groups *n* (% group)				
	Low *N* = 25	Lower middle *N* = 30	Upper middle *N* = 28	High *N* = 36	*p*-value^ǂ^
National strategy or plan	6 (24)	14 (47)	10 (36)	14 (39)	<0.001
Routine surveillance	12 (48)	25 (83)	26 (93)	36 (100)	<0.001
Awareness campaign	7 (28)	18 (60)	13 (46)	15 (42)	<0.001
Prevention in healthcare settings	7 (28)	22 (73)	24 (86)	31 (86)	<0.001
Health worker vaccination	6 (40)	16 (64)	24 (92)	31 (97)	<0.001
	Physician density groups *n* (% group)				
	Low *N* = 19	Lower middle *N* = 28	Upper middle *N* = 34	High *N* = 38	*p*-value^ǂ^
National strategy or plan	4 (21)	11 (39)	16 (47)	13 (34)	<0.001
Routine surveillance	7 (37)	24 (86)	30 (88)	38 (100)	<0.001
Awareness campaign	6 (32)	13 (46)	13 (38)	21 (55)	<0.001
Prevention in healthcare settings	5 (26)	19 (68)	28 (82)	32 (84)	<0.001
Health worker vaccination	3 (27)	18 (75)	25 (83)	31 (94)	<0.001

^ǂ^Mann-Whitney test

### Univariate logistic regression

Results of univariate logistic regression mirrored those found in descriptive analyses. MS were more likely to report having routine viral hepatitis surveillance if they were in the higher binary categories for income level (OR 12; 95% CI 3.9–56), HDI (OR 44; 95% CI 8.4–800), health expenditure (OR 15; 95% CI 4.0–98) and physician density (OR 8.8; 95% CI 2.9–33). Likewise, they were more likely to report having a national strategy and/or policy/guidelines for preventing infection in healthcare settings if they were in the higher binary categories for income level (OR 8.1; 95% CI 3.4–21), HDI (OR 6.3; 95% CI 2.7–16), health expenditure (OR 5.5; 95% CI 2.3–14) and physician density (OR 4.7; 95% CI 2.1–11).

When successively higher quartiles of the four socioeconomic indicators were compared to the lowest quartiles, MS were found to have increasingly higher odds of answering affirmatively to the routine surveillance question and to the healthcare setting prevention question as their quartile rankings increased (Table 2). Odds ratios could not be computed for the highest quartiles for any of the socioeconomic indicators in relation to the routine surveillance question because 100% of the highest-quartile MS in all four cases had routine surveillance.

**Table 2:** Univariate logistic regression producing crude odds ratios for associations between two survey questions and four socioeconomic indicators, by quartile

	N_exposed_ (%)	N_unexposed_ (%)	OR	95% CI
Routine surveillance				
Income level groups				
Low	6 (43)	8 (57)	Ref.	-
Lower middle	25 (74)	9 (26)	3.7	(1.0–14)
Upper middle	30 (91)	3 (9)	13	(3.0–77)
High	38 (100)	0 (0)	∞	∞
Human Development Index groups				
Low	8 (38)	13 (62)	Ref.	-
Lower middle	22 (79)	6 (21)	6.0	(1.8–23)
Upper middle	32 (97)	1 (3)	52	(8.5–1020)
High	37 (100)	0 (0)	∞	∞
Health expenditure groups				
Low	12 (48)	13 (52)	Ref.	-
Lower middle	25 (83)	5 (17)	5.4	(1.7–20)
Upper middle	26 (93)	2 (7)	14	(3.3–100)
High	36 (100)	0 (0)	∞	∞
Physician density groups				
Low	7 (37)	12 (63)	Ref.	-
Lower middle	24 (86)	4 (14)	10	(2.7–47)
Upper middle	30 (88)	4 (12)	13	(3.4–59)
High	38 (100)	0 (0)	∞	∞
Prevention in healthcare settings				
Income level groups				
Low	5 (36)	9 (64)	Ref.	-
Lower middle	17 (50)	17 (50)	1.8	(0.5–7.0)
Upper middle	29 (88)	4 (12)	13	(3.1–67)
High	33 (87)	5 (13)	12	(3.0–55)
Human Development Index groups				
Low	8 (38)	13 (62)	Ref.	-
Lower middle	22 (79)	6 (21)	3.1	(0.9–11)
Upper middle	32 (97)	1 (3)	7.4	(2.3–27)
High	37 (100)	0 (0)	23	(5.7–120)
Health expenditure groups				
Low	7 (28)	18 (72)	Ref.	-
Lower middle	22 (73)	8 (27)	7.0	(2.3–25)
Upper middle	24 (86)	4 (14)	15	(4.3–69)
High	31 (86)	5 (14)	16	(4.7–64)
Physician density groups				
Low	5 (26)	14 (74)	Ref.	-
Lower middle	19 (68)	9 (32)	5.9	(1.7–23)
Upper middle	28 (82)	6 (18)	13	(3.6–56)
High	32 (84)	6 (16)	15	(4.2–63)

### Multivariable logistic regression

In fully adjusted multivariable models, socioeconomic indicator variables continued to not have significant associations with the presence of a national strategy or plan, or with the funding of awareness campaigns. Socioeconomic associations observed for the question about prevention in healthcare settings were no longer significant. In the final (fully adjusted) model, MS in the higher binary HDI category for all of the socioeconomic indicators were more likely than MS in the lower binary category to report having routine surveillance (adjusted OR 26; 95% CI 2.0–340) (Table 3).

**Table 3:** Multivariable logistic regression producing adjusted odds ratios (95% CI) for reporting having a surveillance system by binary socioeconomic indicators, using lower categories as reference groups

	Crude odds ratio (95%CI)	Adjusted odds ratio (95%CI)	*P*-value multivariable model (Wald’s test)
Income level	12 (3.4–46)	0.7 (0.1–9.9)	0.783
Human Development Index	44 (5.6–340)	26 (2.0–340)	0.013
Health expenditure	15 (3.3–69)	1.7 (0.1–36)	0.727
Physician density	8.8 (2.7–28)	2.3 (0.5–9.0)	0.241

## Discussion

This study adds to the growing body of literature on health governance in a global setting [20]. Although the association of individual wealth and health outcomes has been extensively researched [21], our study appears to be unique in that it addresses the potential influence that national socioeconomic indicators have on hepatitis governance and policy. Such a line of inquiry seems important to introduce into the health policy discourse in light of the resource constraints that hobble many aspects of health system functioning in poorer countries. That is, the starting point for our study was the hypothesis that such constraints may inhibit health policy-making. In regard to national responses to viral hepatitis, this study has yielded the intriguing finding that countries with higher and lower measures of socioeconomic status (SES) do not appear to differ greatly regarding their likelihood of reporting the existence of some national policies and programmes. The overall state of the policy response to viral hepatitis during the time period captured in the *2013 Global Policy Report* suggests that urgent action may be needed in many countries; the added value of the current study is that it points to the importance of addressing these issues worldwide without regard for country socioeconomic status.

Most notably, less than half of the 119 WHO MS included in this analysis reported having a written national viral hepatitis strategy or plan, and less than half reported funding viral hepatitis awareness campaigns. The 2014 World Health Assembly resolution on viral hepatitis [7] may have encouraged some governments to give greater attention to viral hepatitis, and it seems likely that more countries have enacted strategies and have funded awareness campaigns since the resolution was approved. What is important for steering the global response to viral hepatitis is to recognise that countries at all levels of socioeconomic development warrant monitoring in these regards, and that all countries might benefit from technical support in relation to developing viral hepatitis strategies and raising public awareness.

In our study, the only association that remained in multivariate analysis was an association between having routine viral hepatitis surveillance and being in the higher binary HDI category. As noted in the methodology section, HDI scores reflect three measures: life expectancy at birth, mean years over expected years of schooling, and gross national income per capita [16]. The study finding thus suggests that a country’s willingness or capacity to implement routine viral hepatitis surveillance is not merely a funding issue. It may be the case that multiple other elements of strong health systems are required in order for governments to consider it feasible to implement routine viral hepatitis surveillance – for example, high-functioning healthcare delivery systems and an educated workforce. If this is the case, then merely providing financial support to lower-income countries to implement viral hepatitis surveillance may be an inadequate strategy for improving the situation, and more complex interventions should be considered.

Contrary to expectation, our results do not show that countries with markers of high socioeconomic status are more likely than other countries to have the selected hepatitis policies in place in any areas other than routine surveillance. These results imply that health outcomes are not simple products of policy and resource availability. Rather, the circumstances that create observed outcomes in health are likely to be more complex and multifaceted. For example, data from the 2016 Hep-CORE policy study in European and Mediterranean countries is consistent with our finding that high-SES countries do not necessarily have stronger policy responses to viral hepatitis. High-income Hep-CORE study countries such as Austria, Denmark and Sweden were reported to not have national written strategies for HBV and/or HCV, whereas middle-income Hep-CORE countries such as Romania, Turkey and Ukraine were reported to have such strategies [22].

Higher prevalence of viral hepatitis in low-SES countries [2, 3] is one possible explanation that should be considered for the failure of higher-income to distinguish themselves with stronger policy responses. That is, the countries with the largest viral hepatitis epidemics may have the greatest incentive to implement policies to effectively address this situation. This study did not seek to identify associations between viral hepatitis prevalence and the enactment of policies because a lack of adequate data from many countries has made it difficult to generate accurate estimates of the HBV and HCV disease burden in the past [2, 3]. The situation is improving, and future studies of factors that affect viral hepatitis policy-making may be able to capitalise on more reliable epidemiological evidence.

With the introduction of WHO’s first-ever *Global Health Sector Strategy on Viral Hepatitis, 2016–2021* in May 2016, the viral hepatitis policy landscape has changed [3]. The strategy is potentially a powerful tool for guiding country-level responses to viral hepatitis, with a focus on its elimination. The four viral hepatitis policy questions that served as dependent variables in our study all reflect major components of the *Global Health Sector Strategy*. Conducting routine surveillance, for example, is essential for countries to acquire reliable data for decision-making. Raising public awareness about viral hepatitis is essential for encouraging the large numbers of undiagnosed people in many countries to come forward for HBV and HCV testing. Uneven implementation of the relevant policies across different countries thus threatens to limit global progress toward the elimination of HBV and HCV. WHO has proposed a monitoring framework to track progress toward *Global Health Sector Strategy* targets, but the framework does not include a policy component [23]. By calling attention to policy gaps in countries at all income levels, our study suggests that it may be beneficial to incorporate policy indicators into the WHO monitoring framework and to encourage policy monitoring in all countries worldwide.

### Limitations

Survey responses from national viral hepatitis focal points were not cross-checked with other sources of information to evaluate their accuracy, and it is therefore possible that some of the policy data utilized in this study are incorrect. Higher response rates from MS above the median HDI and physician density suggest the potential for selection bias. Thus, countries without viral hepatitis programmes are likely to be underrepresented in this study. A lack of response from countries may be attributable to the lack of a clearly designated national focal point. Excluding respondents with missing indicators may have contributed to systematic error. Furthermore, the small sample size may have introduced type II error, meaning that observations of non-significant associations may in fact be false. As this was an ecological study, it is not possible to make inferences at the individual country level. Other socioeconomic or contextual indicators may, in fact, be better predictors of the policies and programmes investigated; indeed, they may even be confounders of the current indicators investigated. In addition, socioeconomic indicators do not measure political commitment to a cause. Countries with a perceived high burden of viral hepatitis may be more likely than other countries to have a political commitment to addressing viral hepatitis. Nevertheless, countries often do not have reliable prevalence estimates available, and are unaware of the actual disease burden and of the importance of implementing strong disease control measures.

## Conclusions

Countries with higher and lower socioeconomic status indicators did not appear to differ greatly regarding the existence of some key national viral hepatitis policies and programmes. This suggests that the policy response to viral hepatitis may not merely depend on national income and other socioeconomic factors. With regard to country-level efforts to reduce and eliminate viral hepatitis, a more nuanced understanding of the multifaceted interactions of socioeconomic factors, health policy, service delivery and health outcomes is needed. Further studies of this nature would benefit from the inclusion of all WHO MS and from the use of additional socioeconomic indicators as well as viral hepatitis prevalence data.

## Abbreviations

CI: Confidence intervals
HBV: Hepatitis B virus
HCV: Hepatitis C virus
HDI: Human Development Index
MS: WHO Member States
OR: Odds ratio
SES: Socioeconomic status
WHO: World Health Organization
